# Supplementary material for: Autophagy-Like Cell Death Regulates Hydrogen Peroxide and Calcium Ion Distribution in Xa3/Xa26-Mediated Resistance to Xanthomonas oryzae pv. oryzae
Source: Int J Mol Sci. 2019 Dec 27;21(1):194. doi: 10.3390/ijms21010194 (PMC6981989; doi:10.3390/ijms21010194)
Supplement: Supplementary file 1 [file ijms-21-00194-s001.pdf]

Article

Autophagy-Like Cell Death Regulates Hydrogen Peroxide and Calcium Ion Distribution in *Xa3/Xa26*-Mediated Resistance to *Xanthomonas oryzae* pv. *oryzae*

Jianbo Cao [1,2,*], Meng Zhang [1], Mengmeng Zhu [1], Limin He [2], Jinghua Xiao [1], Xianghua Li [1] and Meng Yuan [1,*]

1 National Key Laboratory of Crop Genetic Improvement, National Center of Plant Gene Research (Wuhan), Huazhong Agricultural University, Wuhan 430070, China; mengzhang@webmail.hzau.edu.cn (M.Z.); zhumengxv@yeah.net (M.Zh.); xiaojh@mail.hzau.edu.cn (J.X.); xhli@mail.hzau.edu.cn (X.L.)

2 Public Laboratory of Electron Microscopy, Huazhong Agricultural University, Wuhan 430070, China; limin_room@163.com (L.H.)

* Correspondence: myuan@mail.hzau.edu.cn (M.Y.); caojb@mail.hzau.edu.cn (J.C.) Tel.: +86-27-8728-2466

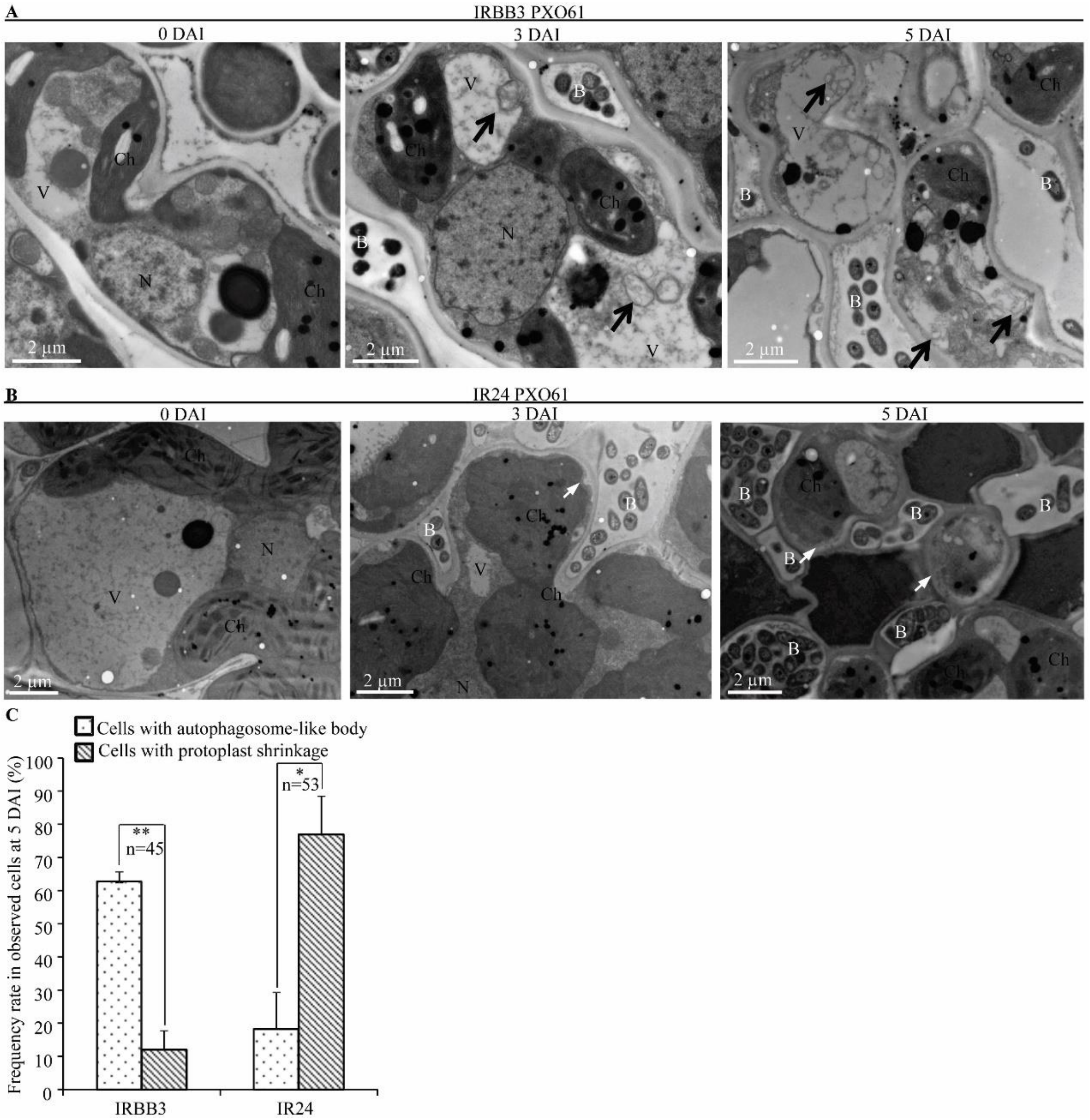

Supplemental Figure 1. Autophagy-like ultrastructure in *Xa3*/*Xa26*-mediated resistance, in comparison with protoplast shrinkage in susceptible reaction in mesophyll cells. Ch, chloroplast; V, vacuole; N, nucleus; B, *Xoo* bacterium; black arrow, autophagosome-like bodies with double membrane; white arrow, protoplast shrinkage. (**A**) Many autophagosome-like bodies in mesophyll cells of IRBB3 plants at 3,5 DAI, in comparison with 0 DAI. (**B**) Many xylem parenchyma cells with protoplast shrinkage in IR24 plants at 3,5 DAI, in comparison with 0 DAI. (**C**) Percentage of cells with autophagosome-like bodies and protoplast shrinkage in mesophyll cells at 5 DAI. Data represent mean (45-53 mesophyll cells of leaf from three different plants) + standard deviation (SD). Double asterisks (**) and single asterisk (*) stand for a significant difference between frequency rate of cells with autophagosome-like body and frequency rate of cells with protoplast shrinkage at $P<0.01$ and $P<0.05$, respectively. n, the number of all the observed cells.

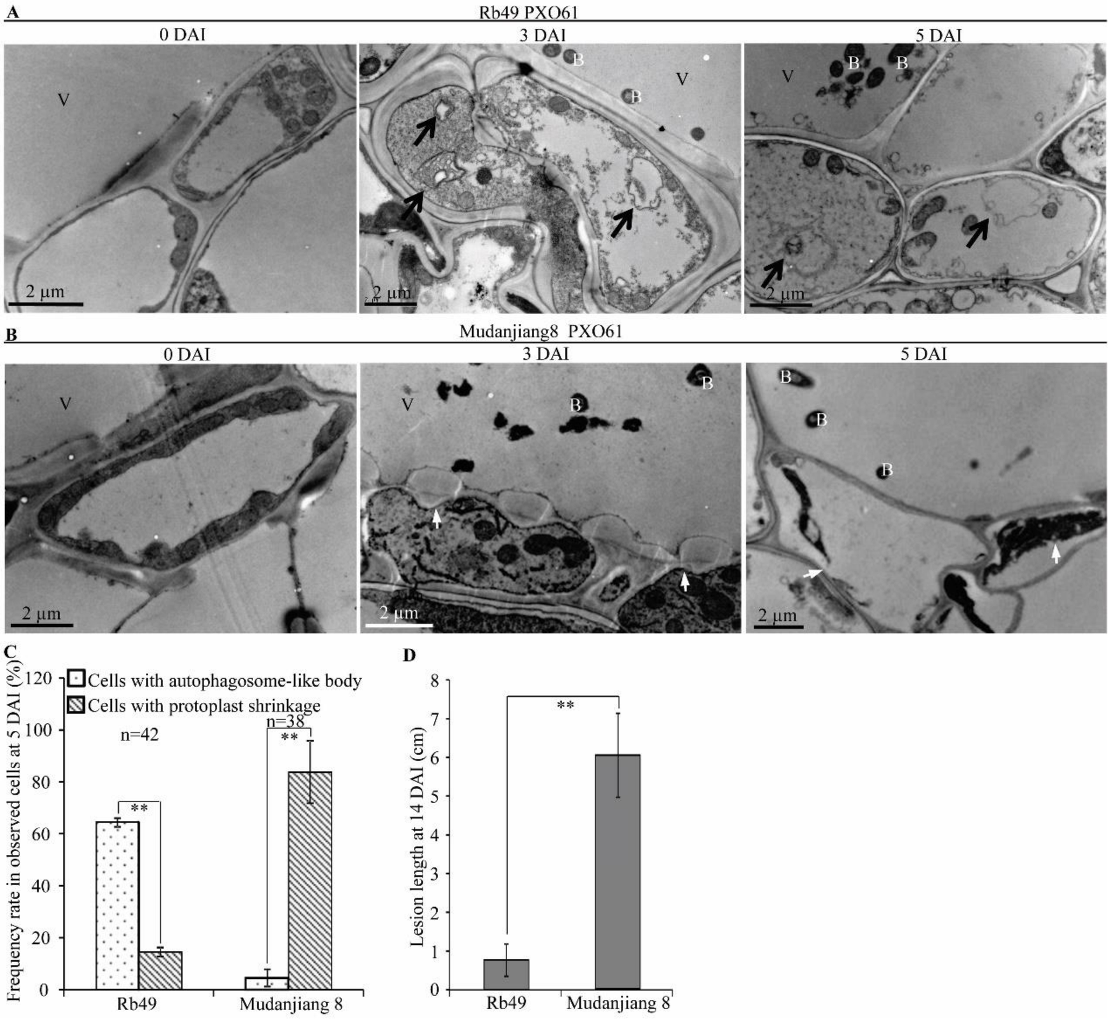

Supplemental Figure 2. Autophagy-like ultrastructure of xylem parenchyma cells in *Xa3*/*Xa26*-mediated resistance, in comparison with protoplast shrinkage in susceptible reaction in *japonica* rice varieties Rb49 and Mudanjiang 8. XV, xylem vessel; B, *Xoo* bacterium; black arrow, autophagosome-like bodies with double membrane; white arrow, protoplast shrinkage. (**A**) A lot of autophagosome-like bodies in xylem parenchyma cells of Rb49 plants at 3, 5 DAI, in comparison with 0 DAI. (**B**) Many xylem parenchyma cells with protoplast shrinkage in Mudanjiang 8 plants at 3, 5 DAI, in comparison with 0 DAI. (**C**) Percentage of cells with autophagosome-like body and protoplast shrinkage in xylem parenchyma cells at 5 DAI. Data present mean (38-42 xylem parenchyma cells of three xylem veins from three different plants) + standard deviation (SD). Double asterisks (**) stand for a significant difference between frequency rate of cells with autophagosome-like body and frequency rate of cells with protoplast shrinkage at $P<0.01$. n, the number of all the observed cells. (**D**) Lesion length of Rb49 and Mudanjiang 8 plants challenged with *Xoo* at 14 DAI. Bars represent mean (13-16 leaves from four plants) + SD. Double asterisks (**) stand for the significant difference between resistant and susceptible plant at $P<0.01$.

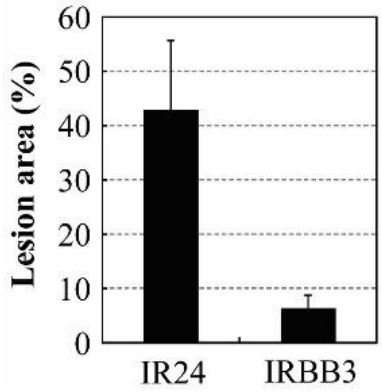

Supplemental Figure 3. Lesion areas of leaves of IR24 and IRBB3 plants from the same batch of qRT-PCR plants at 14 DAI indicate the susceptible reaction and resistant reaction. Bars represent lesion area mean (10-18 leaves from three plants) + standard deviation.

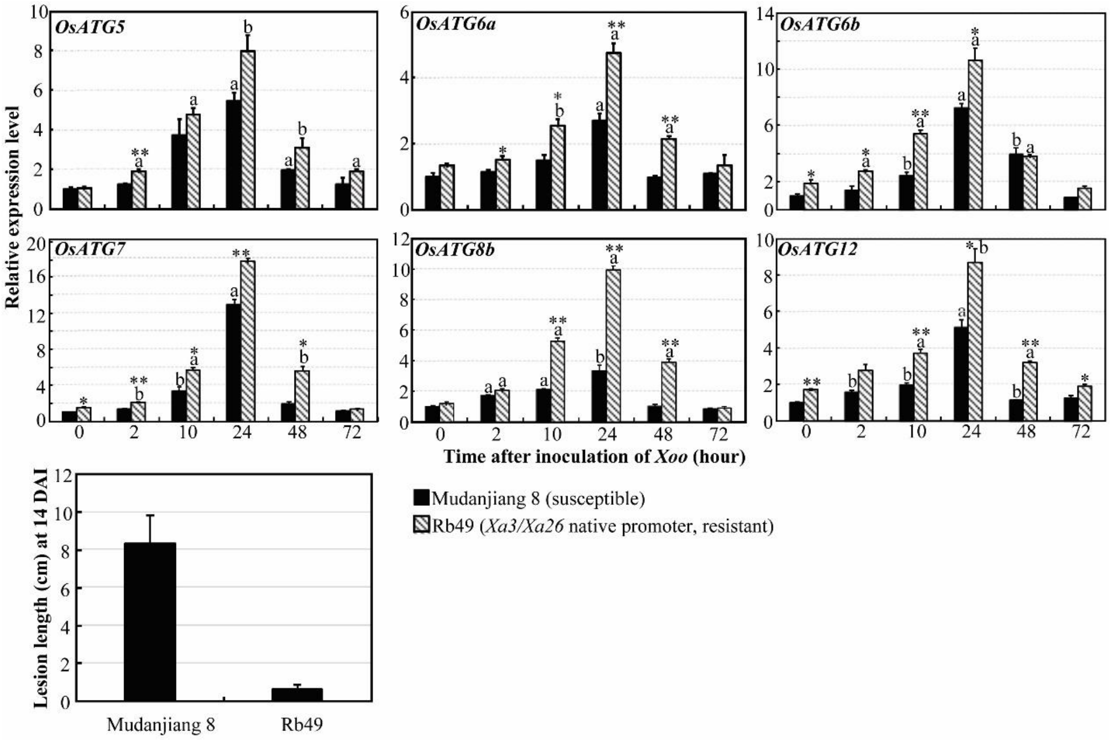

Supplemental Figure 4. Autophagy-related gene (*ATG*) expression level during *Xa3*/*Xa26*-mediated resistance in *japonica* rice varieties. Expression of autophagy-related genes analyzed by qRT-PCR in resistant (Rb49) and susceptible (Mudanjiang 8) rice plants. Rice plants were inoculated with *Xoo* strain PXO61 at four-leaf stage. Data are presented as mean (three replicates) + standard error (SE). Letter "a" and "b" indicate statistically significant differences between 0 h and other time at P<0.01 and P<0.05, respectively in same rice plants. Double asterisks (**P<0.01) and single asterisk (*P<0.05) indicate different levels of statistical significance between Rb49 and Mudanjiang 8 inoculated at same time point. Lesion length of leaves of Mudanjiang 8 and Rb49 plants from the same batch of qRT-PCR plants at 14 DAI indicates the susceptible reaction and resistant reaction, bars represent lesion area mean (17-20 leaves from at least six plants) + SE.

Supplemental Table 1. Percentage of infiltrating inoculation site with water-soaked symptoms influenced by 3-MA in DAB staining.

Plant inoculation after 3 days [1]	water-soaked symptoms Yes (%)[2]	No (%)[3]	N[4] (Inoculation sites)
Rb49 3-MA	0	100	12
Mudanjiang 8 PXO61	100	0	18
Rb49 PXO61+3-MA	**67**	33	36
Rb49 PXO61	**31**	69	42

[1] almost 10^9 *Xoo* strain PXO61 cells in H_2O solution, in 5 mM 3-methyladenine (3-MA) solution and in only 3-MA solution infiltrated into leaves of Rb49 and Mudanjiang 8 plants at four-leaf stage. [2] the percentage of inoculation sites with water-soaked symptom in total infiltrated sites. [3] the percentage of inoculation sites without water-soaked symptom in total infiltrated sites. [4] the number of total infiltrated inoculation sites from at least 3 independent plants.

Supplemental Table 2. Percentage of infiltrating inoculation site with water-soaked symptoms influenced by 3-MA in calcium ion localization.

Plant inoculation after 3 days [1]	water-soaked symptoms Yes (%)[2]	No (%)[3]	N[4] (Inoculation sites)
Rb49 PXO61	**13**	87	30
Rb49 PXO61+3-MA	**85**	15	48
Rb49 3-MA	0	100	24
Mudanjiang 8 PXO61	92	8	24

[1] almost 10^9 *Xoo* strain PXO61 cells in H_2O solution, in 5 mM 3-methyladenine (3-MA) solution and in only 3-MA solution infiltrated into leaves of Rb49 and Mudanjiang 8 plants at four-leaf stage. [2] the percentage of inoculation sites with water-soaked symptom in total infiltrated sites. [3] the percentage of inoculation sites without water-soaked symptom in total infiltrated sites. [4] the number of total infiltrated inoculation sites from at least 3 independent plants.

Supplemental Table 3. PCR primers used for quantitative RT-PCR assays

Gene (GenBank accession number or RGAP code) [a]	Forward primer 5′-3′	Reverse primer 5′-3′	Product size (nt)	Purpose
Os*ATG1a6* (Os03g16130)	CTGACCGCTCACCTGTACAAT	TTTCGCTACTTCTCCCTAATCG	118	qRT-PCR
Os*ATG5* (Os02g02570)	GCCTAAGGATGGTAGATGAAATG	TCCAAGATGAGAACCAAGACC	84	qRT-PCR
Os*ATG6a* (Os01g48920)	TGTCCAAACATCCAATGTGATAAG	TCTGTTCAATGTGCGGCTAC	88	qRT-PCR
Os*ATG6b* (Os03g44200)	AATGCGAGTGATACGAGATCC	GTTGATTGGATATGCTGTTGATTG	89	qRT-PCR
Os*ATG7* (Os01g42850)	AGTCCATCAGACGATGAGAATG	GGTAATGTTTACACTGTGATTTGC	85	qRT-PCR
Os*ATG8b* (Os04g53240)	GACCTACAGTGGCGAGAAC	TGAGAGGATCTTGGCATTACTAC	157	qRT-PCR
Os*ATG8c* (Os08g09240)	AAACCTACCCTGTAATCATCAAAG	CAGAGAGAACACTGAAGCAATG	114	qRT-PCR
Os*ATG9b* (Os10g07994)	ACGGATAATACCTAGAAGCAGTG	GCGTTCACAAGTGTTCCAAC	80	qRT-PCR
Os*ATG10a* (Os04g41990)	ATTTCATGTACCGCTTCACTTG	ATCCGTTCTGACATTCTAGGAG	90	qRT-PCR
Os*ATG12* (Os06g10340)	ATTTACTTGCCGAAACACTCATC	TGCTGCTACTGACATCAACG	103	qRT-PCR
Os*ATG18e* (Os01g57720)	CTGGAGTTCTTGACCTCTCTTC	GTAAGATGGAAACCCAACTTCAC	141	qRT-PCR
Actin (X15865)	TGTATGCCAGTGGTCGTACCA	CCAGCAAGGTCGAGACGAA	121	qRT-PCR

[a]RGAP (Rice Genome Annotation Project, http://rice.plantbiology.msu.edu/) locus identifier.
